# Niagara and Orleans County Veterinary Association

**Published:** 1896-08

**Authors:** J. P. Thomson

**Affiliations:** Secretary


### NIAGARA AND ORLEANS COUNTY VETERINARY ASSOCIATION.

On Monday, July 27, 1896, a meeting of the veterinarians of Niagara and Orleans Counties was called by J. P. Thomson, Secretary of Niagara County, for the purpose of considering the organizing of a County Veterinary Medical Society and also to discuss matters bearing upon the profession in general.

The meeting was called to order at 4 P. M. by J. P. Thomson, Secretary.

Moved and seconded that Dr. Stocking be appointed chairman; carried.

On motion, it was decided to proceed to the election of permanent officers from those veterinarians present; carried. Ballots cast, and Dr. Martin, of Lockport, was elected president. Dr. Stocking, of Medina, was elected vice-president. Moved and seconded that the present secretary, Dr. J. P. Thomson, of Niagara Falls, be considered secretary-treasurer; carried; and that Dr. Kesler, of Holly, Dr. Hunter, of Lewiston, and Dr. Williams, of Middleport, be appointed censors; carried.

Topics relating to the uplifting of the profession, the protection of the community against imposition and the animals against cruelty at the hands of illiterate practitioners, were discussed.

Reports relating to the use of tuberculin and mallein as diagnostic agents, and tetanus antitoxin as a curative agent, were listened to.

The secretary was instructed to have the report of meeting published in the journals for August.

A committee was appointed to formulate constitution and by-laws, to be presented at next meeting, which shall be held at Medina, August 18th, at 2 P. M.

Moved and seconded to adjourn; carried.

J. P. Thomson,
Secretary.

